# Understanding the Barriers and Pathways to Male Help-Seeking and Help-Offering: A Mixed Methods Study of the Impact of the Mates in Construction Program

**DOI:** 10.3390/ijerph16162979

**Published:** 2019-08-19

**Authors:** Victoria Ross, Neil Caton, Jorgen Gullestrup, Kairi Kõlves

**Affiliations:** 1 Australian Institute for Suicide Research and Prevention, WHO Collaborating Centre for Research and Training in Suicide Prevention, School of Applied Psychology, Griffith University, Mount Gravatt 4122, Australia; 2 MATES in Construction, Spring Hill 4000, Australia

**Keywords:** suicide prevention, males, construction industry, help-seeking, help-offering, mixed- methods

## Abstract

The Mates in Construction (MATES) program was developed to address the issue of high suicide rates among males in the Australian construction industry. The program delivers early intervention training and support to construction workers. This mixed-methods study aimed to (1) examine the effectiveness of training for MATES connectors and (2) examine the barriers, motivations and pathways to help-seeking and help-offering for both MATES connectors and clients. A total of 104 volunteers completed a short survey before and after connector training sessions. Quantitative data analysis showed significant increases in connectors’ self-reported suicide awareness, and willingness to offer help to workmates and seek help themselves. For the qualitative component, 27 connectors and clients participated in focus groups and individual interviews. Thematic analysis identified six themes from the connectors’ data: awareness, skills and confidence; removing stigma; making a difference; simplicity of the model; understanding the industry; and visibility, camaraderie and passion. For clients, three key themes emerged: barriers and pathways to help-seeking; speaking the same language; and flow-on effects. The results provide evidence for the effectiveness of connector training and indicate that MATES’s peer support model is enabling workers to overcome traditional barriers and attitudes to seeking and offering help.

## 1. Introduction

There is growing evidence from around the world that males working in the construction sector are in one of the highest occupational risk groups for suicide [1,2]. Mates in Construction (MATES) was developed as a workplace suicide prevention program after research revealed significantly higher suicide rates among Australian men in the construction industry compared to Australian men in general [3]. MATES is a multimodal suicide prevention and early intervention program delivering training and support to workers across a range of areas. Training comprises the following programs:

- General awareness training (GAT) for construction workers—A one-hour session with all workers on a worksite about suicide as a preventable problem faced by the industry, what it looks like when a mate is struggling and how to connect a mate to help;
- Connector training—A four-hour onsite training session for workers who volunteer to become connectors. Connectors are recruited during GAT training (i.e., they tick a box on the training card to self-nominate). The training includes Livingworks safeTALK [4] training. Connectors are trained to identify and safely engage with people at risk and connect them to professional help; and
- ASIST (Applied Suicide Intervention Skills Training)—A 16 h workshop off site for key workers on site (supervisors, union and safety representatives, first aid/site paramedics) where they are trained to make a safe-plan for a person at risk of suicide and connect them to external resources.

Support is also provided to construction workers and their families through field officers (MATES employees who deliver training and support workers on sites), case managers (MATES employees with a minimum qualification of Bachelor/Master of Social Work or Psychology who provide support to workers and connect them to appropriate clinical and nonclinical services) and a suicide prevention hotline. To date, training has been delivered to more than 184,000 construction workers in Australia, and over 8435 workers have received case management assistance.

Given the importance of evaluation in establishing program effectiveness and in providing essential evidence on the strengths and weaknesses of programs, several studies have sought to evaluate the effectiveness of MATES. In a study of over 7000 construction workers, Gullestrup, Lequertier and Martin [5] were able to demonstrate the social validity and general impact of the MATES program, as well as significant increases in suicide prevention awareness in GAT participants compared to a comparison group. In addition, analysis of the Australian National Coroners’ Information System (NCIS) data showed a (non-significant) decrease in male suicides in the Queensland construction industry in the first five years after the introduction of the program compared to the five years before [6]. More recently, GAT training evaluation demonstrated the effectiveness of GAT in shifting beliefs about suicide and mental health [7]. As the impact of training for connectors has not yet been demonstrated, the first aim of the present study was to examine the effectiveness of connector training; specifically, in suicide awareness and knowledge, help-offering and help-seeking.

While it is important to quantitatively determine the effectiveness of MATES, there is also a need for deeper research from a process evaluation perspective to better understand which aspects of the program are working and why. Research into occupational health in males has shown that men are less likely to engage in help-seeking or health promotion behaviours and more likely to conceal mental health issues [8,9,10]. It is therefore critical to better understand construction workers’ barriers, motivations and pathways to receiving help. There is a general lack of qualitative research on male suicide in the construction industry [11], and a knowledge gap exists into how the MATES program impacts clients, as well as the volunteers who provide support. Therefore, the second aim of this study was to address this gap by examining the barriers, motivations and pathways to help-seeking and help-offering of both MATES clients and connectors.

## 2. Materials and Methods

A mixed-methods design was applied to quantitatively examine the effectiveness of connector training, and qualitatively study the experiences and perspectives of both connectors and clients of the MATES program. The study was approved by the Griffith University’s Human Research Ethics Committee (GU Reference number 2017/353).

### 2.1. Connector Training Survey

Volunteers undertaking connector training between May and June 2018 at construction sites across Queensland, Australia, were asked to complete a short paper-based survey. MATES field officers delivering the training administered the survey to 104 participants immediately prior to and immediately after eight separate connector training sessions. The survey consisted of seven items measuring suicide awareness and knowledge, attitudes to help-seeking and help giving; and one question measuring emotional well-being. The first question, *I am familiar with Mates and Construction and the work that they do* was included in the pretraining survey only. The help-seeking item: *If I was going through a difficult time, feeling upset, or was thinking about suicide, I would be willing to seek help* and list of response options (e.g., *intimate partner*, *friend, doctor*) were adapted from the General Help-Seeking Questionnaire [12]. All of the items in the survey required responses on a five-point Likert scale from 1 = strongly disagree, to 5 = strongly agree. The well-being item required participants to select response items regarding how they were feeling emotionally/mentally from 1 to 5, from 1 = very bad, 2 = bad, 3 = OK, 4 = good, 5 = very good. The data from the pre- and post-surveys was entered by MATES administrative staff and the de-identified data file provided to the researchers for analysis.

### 2.2. Interviews and Focus Groups

A total of 27 MATES clients and connectors from Brisbane, Australia participated in individual interviews (n = 10) and focus groups (n = 17) between July and November 2017. All focus groups and individual interviews were digitally recorded and professionally transcribed. All participants were provided with written information, including the identity and affiliation of the researchers, aims of the research and confidentiality and informed consent issues, including the right to withdraw voluntarily. All participants were required to provide written consent before taking part in the research.

#### 2.2.1. Connectors

MATES field officers approached and recruited the connectors and arranged times for the focus groups. The groups were conducted by a researcher/trained facilitator, who also obtained informed consent from participants. There were five focus groups, each comprising three to four connectors, with an overall total of 17 participants (all male). The focus groups followed a semistructured format, where participants were asked about their motivations for becoming connectors, how they used the skills from the MATES training, barriers and enablers to help-offering and help-seeking and the positive and negative aspects of their roles as connectors. The groups were of approximately 45 min duration.

#### 2.2.2. Clients

Clients who had received assistance and/or referrals through the program were identified and contacted by their case managers who invited them to participate in the study. Inclusion criteria were limited to individuals for whom it has been more than six months since receiving assistance from MATES and who were deemed by their case managers to not currently be at risk of suicidality. Contact details of clients who indicated their agreement to participate were provided to researchers who arranged individual interviews, either face-to-face or over the telephone. Given the sensitive nature of the topic, the interview format was considered the most appropriate to ensure clients’ privacy and confidentiality. Of the 10 case management clients who participated in the study, there were eight male construction workers who had directly received assistance from MATES and two female partners of construction workers who had obtained assistance for their partner or as a couple. A semistructured interview (of approximately 30 min) was conducted where participants were asked about their motivations for seeking help through MATES, how they came into contact with MATES, other help-providing services they were aware of at the time, barriers and enablers to help-seeking and how the MATES program and volunteers were or were not helpful.

### 2.3. Statistical Analysis

#### 2.3.1. Connectors’ Survey

All data were analysed using the SPSS 25 statistical package [13]. Wilcoxon signed rank tests were conducted on each of the pre–post items (suicide awareness and knowledge, help-seeking and help giving and emotional well-being). Reliability analysis demonstrated moderate reliability for the pre-training items (excluding, *I am familiar with Mates and Construction and the work that they do*) (α = 0.75) and post-training items (α = 0.71).

#### 2.3.2. Interviews and Focus Groups

A generic qualitative approach was applied separately to the focus group and the interview data, using thematic analysis, a method for identifying and analysing themes within the data [14]. In the first phase of the analysis, two researchers, VR and KK, worked independently, reading and re-reading transcripts, note-taking and applying an inductive approach so that coding and theme development were directed by the content of the data. Next, to ensure validity of analysis, the researchers worked together, reassessing themes and interpretations with any discrepancies negotiated until consensus was reached. This iterative revision process was used to create the final list of themes with supporting verbatim examples from the transcripts. Thematic analysis was conducted separately for the connectors and clients.

## 3. Results

### 3.1. Connectors’ Survey Results

Pairwise deletion was employed for cases that did not provide information for either the pre- or post-measures or both. Final numbers for each of items are shown in Table 1 and Table 2. The difference scores were approximately symmetrically distributed, as assessed by a histogram with a superimposed normal curve. Thus, Wilcoxon signed rank tests were conducted to determine the effect of the connectors’ training and showed a significant increase in the median score for each of the six suicide awareness, help-seeking and help-offering items. A Wilcoxon signed rank test also showed a significant improvement in how participants felt emotionally/mentally after the training. (Table 1).

Wilcoxon signed rank tests were also conducted to examine the effectiveness of connectors’ training on help-seeking intentions (Table 2). Help-seeking intentions significantly increased from before to after training for intentions to seek help from one’s intimate partner, friend, close family, workmate, supervisor, doctor, mental health professional, telephone helpline, MATES worker/connector and minister/religious leader. There was no significant change for intentions to seek help from ‘another’ (i.e., not listed in the response options) or to not seek help from anyone at all.

### 3.2. Focus Grsoups and Interview Results

#### 3.2.1. Connectors’ Perspectives

Thematic analysis identified six key themes from the connectors’ focus group data: awareness, skills and confidence; removing stigma; making a difference; simplicity of the model; understanding the industry; and visibility, camaraderie and passion.

***Awareness, Skills and Confidence***

Connectors reported being initially shocked to learn of the high suicide rates in the construction industry and of how they were previously not aware of the magnitude of the problem. They spoke of the impact of being presented with suicide statistics, and of the importance of having awareness of the issue, and in particular how learning about high construction industry suicide rates was a motivator to continue training, which in turn provided them with the skills and confidence to assist those in need.

There was positive feedback from connectors about the value of learning the skills to identify if someone is experiencing personal difficulties. Participants spoke of how connector training provided them with the confidence to be able to detect if something is wrong (e.g., listening carefully; picking up on body language and emotions) and to speak to a suicidal person and offer assistance. A number of participants indicated that prior to training, they were concerned that they did not know how to help someone in need.

*I had very old-fashioned views about suicide and people - probably not the most supportive. The training brought me right out of that… and really made me realise how in general terms someone would get to a position like that and how successful help could be at the right times if people were keeping an eye out for each other.*

*That concerned me that I didn’t know what I was looking for. It made a lot of sense to me after doing the course. One of the blokes in particular was showing a lot of those symptoms that they were talking about. We could have quite easily missed it.*

***Removing Stigma***

Connectors discussed how that they felt their training was effective in gradually removing the stigma of suicide within the construction industry. They described how through the MATES program, talking about suicide had started to become acceptable, whereas in the past, this was seen as taboo. Several people also mentioned how it was extremely important to learn that it is beneficial, rather than dangerous to ask a potentially suicidal person if they are considering suicide.

*We’ve educated people to the extent that it isn’t a weakness. Everybody suffers and they go through problems. It’s about solving the problem, not making it worse, and get people talking about it then and say ‘do you know what, we’re not bulletproof. We like to think we bloody are, but we’re not’.*

*I feel like the best part of the whole course was the removing the taboo kind of thing. I often thought that if I’m directly asking the question (are you suicidal?), it would be the wrong thing to do. I thought it would be a terrible thing because it would put it in their head and they might think about it, but I found out it’s the best thing to do.*

***Making a Difference***

Many connectors spoke about the positive effects of knowing they had helped someone. Numerous examples were provided of how they had been able to assist others in need and provide a positive contribution to their workplace. In addition, connectors spoke about how they were able to use their MATES training to help people outside of their workplace.

Several connectors pointed out that there were times when workers were not always receptive to help-offering, although this was reported to be rare and usually the case where the worker was using illicit drugs. It was highlighted that although MATES training emphasises that connectors should not feel guilty if unable to help someone, it would be useful to have more opportunities to ‘debrief’ with MATES staff and volunteers.

*I had a member one day call me and tell me that he was thinking about jumping off a building that he was working on. I was able to go to the site and spoke with him and connected him up with some help and he got the counselling and moved forward, which was pretty powerful stuff.*

*The fact that you are actually able to have discussions with people and they feel like you’re taking an interest in them as a person... It’s a two-pronged benefit. The fact that you both walk away from the situation feeling that things are in a better direction and the person’s gone ‘he cares about me, rather than just the name on the shirt’, is the biggest benefit out of it.*

***Understanding the Industry***

Connectors highlighted the importance of MATES being built into the culture of the construction industry, and as such, workers can relate to and identify with MATES. This was said to be particularly the case when it comes to help-seeking. They described how, as a male-dominated industry, they found male construction workers were more comfortable talking to other workmates than calling a general helpline. They also emphasised how GAT training was ‘pitched at the right level’, that is, specifically for construction workers rather than for office workers, without too much focus on psychology/mental health. Connectors also spoke of how MATES was well supported within the construction industry and had united the industry in promoting the mental health of construction workers.

*I know you’ve got any number of other organisations that do it, but MATES, they’re a part of us. They’re a part of the construction industry, so there’s that connection with them. Blokes will identify with that rather than calling (a helpline).*

*The whole thing’s supported by our industry and it’s something that we put together, and everyone pays into, and it’s represented very well. It’s taken off very well.*

***Simplicity of the Model***

Connectors spoke of how they liked the simplicity of the MATES model, which they said made it easy to implement and enable both help-offering and help-seeking. In addition, participants stressed another effective aspect of the MATES model was the clearly defined roles for volunteers, emphasising that they are not mental health workers nor there to ‘fix’ problems. Rather, MATES training provides the skills to recognise when someone needs help, and to be able to connect the person to assistance.

*You’ve got your first aiders on your wall, and your mental health first aiders. They’re two different people. You’re going to him for a cut on the finger: well you go to him for a cut on your heart. I looked at that and I thought ‘Of course. That’s just so simple!’*

*We’re construction workers. We’re not trained mental health professionals - we’re just connectors. We’ll get you from here to there and keep you safe for that bit, and then you’re handing someone over to get the proper help that they need because we can’t fix the problems. We can only help them get the help they need.*

***Visibility, Camaraderie and Passion***

Connectors stressed that MATES’s high visibility on sites, as well as their passion and engagement with workers was integral to the success of MATES. They spoke of what they saw as ‘a huge camaraderie around MATES’ and described how representatives such as field officers were very popular with workers on site, thus making them more approachable. In general, volunteers considered that all of the above factors were integral to MATES’s success.

*It’s the one thing that MATES have done by doing that model is with construction workers if it’s in front of you, you tend to rely on it more so. The fact that the field officers are around the projects and drop in quite frequently, it’s front of centre, front of mind. That reference point is always there.*

*They’re coming from a place of—we can tell they’re not just a contract trainer in to deliver something that they couldn’t give two (expletives) about. It’s a passion and it comes across in their delivery. People can’t not pay attention when someone is delivering like that.*

#### 3.2.2. Clients’ Perspectives

Three key themes were identified: barriers and pathways to help-seeking; speaking the same language; and flow-on effects.

***Barriers and pathways to help-seeking***

The issue of male attitudes, including traditional views such as stoicism and the importance workers place on being the ‘provider’ and workplace culture, were raised as key obstacles to help-seeking within the construction industry. Clients described how they (or in the case of the female interviewees, their male partners) viewed asking for help as a weakness. Several clients also highlighted their extreme reluctance to visit a doctor or health practitioner.

*I know with a lot of men, particularly in that sort of industry, they’ve got a macho image that they’re supposed to uphold. They do find it very difficult to ask for help. They think it’s a weakness and people are going to judge them, which is quite sad.*

[Female partner of a construction worker]

*I had to learn that it’s okay to admit that you’re having trouble and it’s okay to ask for help. That’s an attitude. An attitude stopped me from doing it earlier. A change in attitude helped me get there.*

When speaking about their motivations to first seek help and how they overcame barriers to help-seeking, clients echoed connectors’ perspectives in terms of the importance of high visibility and promotion of MATES on construction sites, which they described as fundamental to their awareness that help was available. Significantly, several interviewees spoke of how they were able to relate to stories from MATES delegates and male peers about help-seeking experiences, which enabled them to move past traditional barriers to reach out for help.

*Our delegate got up and spoke to us … explaining how he was in a bad situation when he was younger… He ended up reaching out, and if he never reached out, who knows where he would have been. I was in a really bad situation, and having someone that you look up to, talk about his own experience… it makes you feel a lot more comfortable. It wasn’t long after that, I ended up reaching out, which was a good thing.*

*I went to a friend’s funeral… we were at the wake afterwards and one of the toughest guys I’ve ever met spoke to me about his experience with suicidal thoughts. That was not long after the (GAT) training. I just went ‘wow, so it doesn’t matter how tough you are on the exterior, everyone’s got feelings and emotions and if you don’t deal with them, they’ll deal with you’. There’s people out there that can help you deal with them.*

***Speaking the Same Language***

A strong overlap was also seen between connectors’ and clients’ viewpoints in their perceptions of MATES as being a part of, and thus having an understanding of the construction industry. Clients reported that MATES workers understand the problems that are unique to those working in the construction industry and that they ‘speak the same language’. Also echoing the connectors, clients clearly expressed their preference to seek help from a service within the construction industry, rather than a mainstream service provider. Clients described feeling relief in discovering that they were not alone and that others have been through similar situations. Although one client reported that it took some time to organise an appointment with a counsellor, clients in general expressed surprise that help was so easily and quickly accessible. MATES’ prompt process for connecting clients to help, regular contact, and call-back and follow-up services were identified by clients as important aspects in how the MATES program had helped.

*Just knowing that someone that you’re talking to has gone through the same thing that you’re going through and that you’re not the only person in the world that feels that way. That gave me a big sense of relief. Those people speak your language and it becomes even more and more real and more understandable.*

*He said ‘mate, you can call this number 24 h a day’. That gave me the feeling that if I’m having trouble at that moment and I don’t know, it could be three o’clock in the morning, I’ve got someone to call. That made me feel good. I reckon that’s a real bonus. You just want someone to talk to when you’re upset. I reckon that’s gold.*

***Flow-on Effects***

Clients also spoke of their positive outcomes from receiving assistance from MATES volunteers. They also described the flow-on effects from their own experiences, such as increasing their openness to help-seeking, as well as having more awareness of other peoples’ problems and openness to helping others.

*I’m personally a lot more open these days to talking about things and I suppose reaching out to people who might be able to help if I think that’s what I need; a lot more open-minded to the fact that it doesn’t make you any less of a person… All that stuff—‘the big tough man’.*

*He talks highly about them (MATES), and now when he has a mate in trouble at work he always goes ‘give these guys a call, even if you just need a chat’.*

[Female partner of a construction worker]

## 4. Discussion

To the best of our knowledge, this mixed-methods study was the first to apply a qualitative approach to examine the barriers, motivations and pathways to help-seeking and help-offering in construction workers. In line with previous positive outcomes from GAT evaluation research [7], results from the connector training survey showed significant increases in connectors’ self-reported suicide awareness, and willingness to both offer help to workmates and seek help themselves. It is important to note; however, that despite statistically significant increases post training, connectors’ levels of suicide awareness and willingness to offer help were already quite high prior to training. This is likely due to the fact that connectors have previously undertaken GAT training and have demonstrated their willingness to help others by volunteering to be connectors. By contrast, their levels of help-seeking were lower at baseline, indicating a more obvious shift in attitudes pre- and post-training.

The connectors’ focus groups provided strong support for these results; qualitatively complementing the findings with underlying reasons for how the MATES training had been effective. Connectors described how having awareness of the problem of suicide in the construction industry and learning skills and gaining confidence in how to speak to a suicidal person had motivated them to help their workmates. Connectors reported that they believed MATES training was helping to gradually reduce the stigma of suicide in the industry, which in turn was helping construction workers to understand that asking for help should not be seen as a weakness. Another important insight gained from connectors was that the simplicity of the MATES model and the clarity of their roles made it easy to implement and facilitate help-offering and help-seeking. It was important to them that their roles were not seen as mental health workers to ‘fix peoples’ problems’ but rather to keep workers safe and connect them to help. Connectors did, however, point out that workers were not always receptive to receiving help and that more opportunities for debriefing with other volunteers would be helpful.

Results showed a significant increase in mean scores on self-reported well-being for connectors post-training. It is plausible that the awareness and confidence gained from the connector training sessions may have contributed to these feelings, as altruistic emotions and behaviours have been found to be associated with greater mental health and well-being [15,16]. This indeed appeared to be the case when connectors spoke of their positive feelings and flow-on effects when they had been able to help someone and ‘make a difference’.

Qualitative data on MATES clients’ perceptions and experiences also provided some extremely valuable insights into our current understanding of how the MATES program is working. Consistent with the literature on traditional masculine attitudes as barriers to help-seeking [9,10,17,18], clients spoke of how their perceptions of males needing to be self-reliant, bullet-proof, ‘the provider’, and viewing help-seeking as a weakness were obstacles to obtaining seeking help. Importantly, some clients described how they were able to personally overcome these barriers and, in particular, how hearing male peers speak about their own help-seeking experiences had given them the confidence to reach out for the help that they needed. In addition, clients spoke of positive outcomes since receiving assistance; their increased awareness of others’ problems and increased openness to help-seeking and help-offering.

Results also indicated considerable overlap in some of the perspectives of connectors and clients. Perceptions of MATES as part of the construction industry and their high visibility on sites were considered as integral to the success of the program by both connectors and clients, demonstrating the strength of these features of the program. Importantly, the perception that MATES staff and volunteers ‘speak the same language’ and understand the problems that are unique to working in the industry were considered fundamental to the success of MATES by both groups.

A clear limitation of this study was the potential for selection bias in the qualitative sample. There were very few negative comments about the MATES program, and this may have been due to potential inadvertent bias of case managers and field officers in selecting participants who had positive experiences with MATES. In addition, the before-and-after training design for the connectors did not enable measurement of the long-term impact of training. The potential confounding factor of response shift bias in self-report studies [19] should also be considered, and the application of an approach such as the retrospective pretest method [20] is recommended to control for this. Future research would also benefit from randomly selected samples to ensure an accurate representation of construction workers, as well as the inclusion of follow-up connectors’ data. Other recommended approaches to future research are the application of a randomised, controlled trial and comparisons between age groups on barriers to help-seeking and help-offering.

## 5. Conclusions

The results indicate the effectiveness of MATES connector training in improving suicide prevention awareness, and help-offering and help-seeking in connectors. It is encouraging that the program appears to be enabling workers to overcome traditional barriers and attitudes to help-seeking through the positive stories of seeking/receiving help from industry peers. These findings suggest it will be critical for MATES to continue to their focus on the peer support model, both to encourage help-seeking and offering and to continue to reduce stigma of mental health and suicide in the construction industry.

## Figures and Tables

**Table 1:** Suicide awareness: pre- and post-connectors’ training.

Suicide Awareness Items and Well-Being		Pre-Training					Post-Training						
	*N*	Me ^1^	Md ^2^	*Mo* ^3^	*Range*	*SD*	Me ^1^	Md ^2^	*Mo* ^3^	*Range*	*SD*	*Z* ^4^	*p*
**Suicide awareness** *	91	4.31	4.33	4.67	2.33–5	0.54	4.71	4.83	5	3–5	0.37	−7.12	<0.001
1. I am familiar with MATES in Construction and the work that they do.	93	4.49	5	5	1–5	0.72	-	-	-	-	-	-	-
2. Talking openly about suicide can prevent suicide.	94	4.54	5	5	1–5	0.70	4.85	5	5	4–5	0.36	−4.19	<0.001
3. If my workmate was going through a difficult time feeling upset or thinking about suicide, I think I would notice.	94	3.90	4	4	1–5	0.93	4.55	5	5	1–5	0.67	−6.05	<0.001
4. If my mate was going through a difficult time feeling upset or was thinking about suicide, I would be willing to offer help.	94	4.78	5	5	3–5	0.47	4.89	5	5	4–5	0.31	−2.52	0.01
5. If my workmate was going through a difficult time feeling upset or thinking about suicide, I would know how to connect him/her to appropriate help.	94	4.03	4	4	1–5	0.92	4.82	5	5	1–5	0.53	−6.84	<0.001
6. My current worksite supports good mental health and well-being.	91	4.32	4	5	2–5	0.79	4.62	5	5	2–5	0.63	−4.19	<0.001
7. If I was going through a difficult time, feeling upset, or was thinking about suicide, I would be willing to seek help.	94	4.29	5	5	1–5	0.89	4.59	5	5	1–5	0.75	−3.91	<0.001
**Well-being**													
So far today, the best way to describe how I’m feeling emotionally/mentally is…	88	4.28	4	5	2–5	0.77	4.40	5	5	2–5	0.70	−2.24	0.03

Note. * Average score of items 2–7. ^1^ Mean. ^2^ Median. ^3^ Mode. ^4^ *Z*-value is based on negative ranks.

**Table 2:** Help-seeking intentions: pre- and post-connectors’ training.

Help-Seeking Sources		*Pre-Training*					*Post-Training*						
	*N*	Me ^1^	Md ^2^	*Mo* ^3^	*Range*	*SD*	Me ^1^	Md ^2^	*Mo* ^3^	*Range*	*SD*	*Z*	*p*
Intimate partner	94	4.28	5	5	1–5	.93	4.44	5	5	1–5	.93	−2.79	0.005
Close family	92	4.08	4	5	2–5	.10	4.32	5	5	2–5	.89	−3.43	0.001
Friend	94	4.00	4	5	1–5	1.02	4.27	4	5	1–5	.87	−3.50	<0.001
Workmate	94	3.40	3	3	1–5	1.14	3.85	4	5	1–5	1.05	−4.88	<0.001
Supervisor	93	2.99	3	3	1–5	1.23	3.44	3	3	1–5	1.27	−4.59	<0.001
Doctor	92	3.71	4	5	1–5	1.32	3.95	4	5	1–5	1.23	−3.66	<0.001
Mental health professional	92	3.92	4	5	1–5	1.21	4.16	5	5	1–5	1.10	−3.32	0.001
Telephone helpline	93	3.26	3	3	1–5	1.29	3.84	4	5	1–5	1.17	−5.30	<0.001
MIC Worker/Connector	91	3.87	4	4	1–5	1.01	4.42	5	5	1–5	.80	−5.75	<0.001
Minister/Religious leader	91	1.79	1	1	1–5	1.15	2.04	1	1	1–5	1.28	−3.08	0.002
Not seek help from anyone	92	1.88	1	1	1–5	1.15	1.85	1	1	1–5	1.28	−0.51 ^4^	0.61
Seek help from another	71	2.39	3	1	1–5	1.33	2.38	3	1	1–5	1.31	−0.07 ^4^	0.95

Note. ^1^ Mean. ^2^ Median. ^3^ Mode. ^4^ These *Z*-values are based on positive ranks, while all other *Z*-values are based on negative ranks.
